# Diagnosis and Therapeutic Management of Liver Fibrosis by MicroRNA

**DOI:** 10.3390/ijms22158139

**Published:** 2021-07-29

**Authors:** Tomoko Tadokoro, Asahiro Morishita, Tsutomu Masaki

**Affiliations:** Department of Gastroenterology and Neurology, Faculty of Medicine, Kagawa University, Kagawa 761-0793, Japan; tadokoro.tomoko@kagawa-u.ac.jp (T.T.); tmasaki@med.kagawa-u.ac.jp (T.M.)

**Keywords:** liver fibrosis, liver cirrhosis, microRNA, exosomal miRNA

## Abstract

Remarkable progress has been made in the treatment and control of hepatitis B and C viral infections. However, fundamental treatments for diseases in which liver fibrosis is a key factor, such as cirrhosis, alcoholic/nonalcoholic steatohepatitis, autoimmune hepatitis, primary biliary cholangitis, and primary sclerosing cholangitis, are still under development and remain an unmet medical need. To solve this problem, it is essential to elucidate the pathogenesis of liver fibrosis in detail from a molecular and cellular perspective and to develop targeted therapeutic agents based on this information. Recently, microRNAs (miRNAs), functional RNAs of 22 nucleotides, have been shown to be involved in the pathogenesis of liver fibrosis. In addition, extracellular vesicles called “exosomes” have been attracting attention, and research is being conducted to establish noninvasive and extremely sensitive biomarkers using miRNAs in exosomes. In this review, we summarize miRNAs directly involved in liver fibrosis, miRNAs associated with diseases leading to liver fibrosis, and miRNAs related to complications of cirrhosis. We will also discuss the efficacy of each miRNA as a biomarker of liver fibrosis and pathology, and its potential application as a therapeutic agent.

## 1. Introduction

The liver can be damaged by viral and parasitic infections, nonalcoholic steatohepatitis (NASH), heavy alcohol consumption, or autoimmune mechanisms, and chronic damage leads to fibrosis and cirrhosis [1]. Cirrhosis is the most common endpoint of chronic liver disease, and like hepatocellular carcinoma (HCC), is associated with a high mortality rate. Complications of cirrhosis, such as gastrointestinal bleeding, ascites, portal vein thrombosis, and hepatic encephalopathy (HE) due to portal hypertension, can significantly impair a patient’s quality of life and ultimately lead to fatal outcomes. In uncompensated cirrhosis with complications, symptomatic treatment is generally the only option. Notably, liver failure and gastrointestinal bleeding are the leading causes of death in patients with cirrhosis. In recent years, it has become possible to control hepatitis virus infections and clinically reverse fibrosis, which is generally irreversible in advanced cases. Therefore, early diagnosis and further elucidation of the molecular mechanisms of liver fibrosis are necessary to develop specific therapies. Liver biopsy, the gold standard for the diagnosis of liver fibrosis, poses several challenges, including invasiveness and the risk of serious complications such as bleeding, sampling errors, variability in histopathological interpretation, and economic costs. It is also not suitable for diagnosing the early stages of cirrhosis. Hence, there is a need for alternative non-invasive diagnostic tests. The search for these tests has led to the development of alternative methods, such as image inspection (e.g., elastography), biochemical scoring (e.g., aspartate aminotransferase-to-platelet ratio index (APRI) and Fib-4 index), and the use of direct markers, such as hyaluronic acid, type IV collagen, Mac-2-binding protein glycosylation isomer, (M2BPGi), and autotaxin [2,3,4]. However, the efficacy of these biomarkers depends on the disease state, and in some instances, diagnostic performance is only moderate [2].

Activation of hepatic stellate cells (HSCs) is a pivotal event in liver fibrosis [5], and various inflammatory and fibrotic pathways are involved [6]. Activated HSCs are precursors of myofibroblasts that produce extracellular matrix (ECM) in the liver. Therefore, the treatment of hepatic fibrosis requires the prevention of hepatocellular damage and control of activated HSCs [7]. If HSC inactivation can be induced by cell- or target-specific pharmacological interventions, then, more effective, less toxic, and more accurate anti-fibrotic treatments than the currently available ones can be developed.

In recent years, research on organ fibrosis has rapidly evolved from classical pathological observations to molecular and cell biological approaches. The molecular mechanism underlying liver fibrosis is associated with non-coding RNAs (ncRNAs). MicroRNAs (miRNAs) are short ncRNAs that are involved in the epigenetic regulation of intracellular and extracellular signaling pathways and the post-transcriptional regulation of genes [8,9]. Furthermore, the effects of miRNA-mediated intercellular signaling mechanisms on liver fibrosis are gradually becoming clearer. Therefore, miRNAs are currently being studied for the diagnosis and monitoring of liver fibrosis and combining them with existing fibrosis scores may further improve diagnostic accuracy [10]. Additionally, extracellular vesicles (EVs), including exosomes, have been attracting attention as intercellular communication media for functional RNAs [11,12]. We will review the use of miRNAs in the pathology and diagnosis of hepatic fibrosis and the development of new therapeutics.

### 1.1. Liver Fibrosis and Cirrhosis

Cirrhosis is the end stage of a variety of chronic liver diseases. It affects 1%–2% of the world’s population and kills more than one million people per year worldwide [13,14]. Many types of cells, cytokines, and miRNAs have been implicated in the initiation and progression of cirrhosis. In particular, the activation of HSCs is a pivotal event in fibrosis [15]. Moreover, Kupffer cells activated by viral infections and other factors attack hepatocytes and promote HSC activation. Repeated apoptosis and regeneration of hepatocytes, mainly HSCs, is the cause of liver cirrhosis.

HSCs, also known as Ito cells, are fat-storing cells containing vitamin A which are found in the space of Disse and account for approximately 10% of the total number of liver cells. HSCs correspond to pericytes that surround the sinusoidal endothelial cells with branch-like projections and contact hepatocytes [16]. HSCs have also been suggested to serve as antigen-presenting cells in the liver, presenting lipid antigens to CD1-restricted T lymphocytes such as natural killer T (NKT) cells and promoting NKT cell proliferation by presenting hepatic NKT cell lipid antigens via interleukin (IL)-15 [17].

HSCs and periportal fibroblasts are the main producers of ECM [5]. ECM deposition is a wound-healing response that protects residual tissue from epithelial cell death, inflammatory cell infiltration, and local proteolytic enzymes. It is reversible when the reaction is mild and terminated, but if the inflammation becomes chronic, the activated cells become myofibroblasts which amplify fibrosis and play a major role in the regulation of inflammation, immune responses, and the HCC microenvironment. At the molecular level, many cytokines are involved in mediating the signaling pathways that regulate HSC activation and fibrogenesis. Pro-inflammatory cytokines, such as platelet-derived growth factor (PDGF), transforming growth factor (TGF)-β, tumor necrosis factor (TNF)-α, and IL-1, shift HSCs from a quiescent state to an activated state [15]. Two types of PDGF, PDGF-BB and PDGF-AB, are important cytokines that promote the proliferation of activated HSCs in liver fibrosis. Notably, the dimeric cellular receptor, PDGF receptor β, is strongly upregulated in activated HSCs [18]. HSCs are activated and transformed into myofibroblast-like cells during liver injury [19], and they proliferate and contribute to inflammatory reactions and overproduce ECM while producing their own fibrosis-inducing factors, such as TGF-β [20].

### 1.2. MicroRNA and Exosomal miRNA

The protein-coding DNA region is estimated to account for less than 2% of the entire human genome, yet approximately 80% of the human genome has some functional activity. Although most of the human genome consists of DNA regions that do not encode proteins, some of these regions produce functional RNAs with a variety of functional activities. These functional RNAs can be divided into two groups: (i) small ncRNAs with less than 200 bases, including miRNAs, and (ii) long ncRNAs with more than 200 bases [21,22]. Small ncRNAs (miRNAs) are small molecules of approximately 22 nucleotides that play an important role in the regulation of gene expression. miRNAs bind complementarily to the 3′ untranslated regions of the target gene and inhibit protein production by cleaving or inhibiting the function of messenger RNA (mRNA). On the other hand, miRNAs have also been reported to interact with other regions, such as the 5’ untranslated regions, and under certain conditions, miRNAs can also activate translation [23]. miRNAs exert their function by binding to Argonaute proteins to form silencing complexes [24]. They regulate a variety of signal transduction processes, including cell proliferation and apoptosis, and inflammatory and fibrotic processes, and are involved in the pathogenesis of many diseases, including cancer [25,26,27,28,29,30,31,32]. For liver diseases which require early diagnosis for determining treatment guidelines but do not have established diagnostic methods or appropriate biomarkers as yet, there is a need for solutions to these challenges. The fact that miRNAs are stably present in body fluids such as blood, urine, and saliva, which are easily collected from living organisms, makes miRNAs suitable as biomarkers.

EVs have attracted attention as delivery media for functional RNAs, including miRNAs. Exosomes are a subset of EVs which are secreted from the endoplasmic reticulum in cells with a lipid bilayer membrane with an average diameter of 30–100 nm [33,34]. They contain mRNAs and miRNAs which they transmit between cells [33]. The endoplasmic reticulum-derived EVs, including exosomes, contain RNA, proteins, and other information from the donor (EV–secreting) cell, and transmit this information to the recipient cell. miRNAs are mostly contained in EVs and are transported to recipient cells by intercellular transport, affecting signal transduction in recipient cells and cell phenotype. miRNAs in EVs extracted from patients are useful as disease biomarkers for various diseases [33]. In addition, a new drug delivery system that hijacks exosomes to deliver anti-miR oligonucleotides into the cells that receive them has been developed and is expected to have therapeutic applications [35].

Furthermore, it is expected to be applied to the diagnosis of liver fibrosis and to the development of novel therapeutic agents using miRNAs.

## 2. Epigenetic Changes in Liver Fibrosis/Cirrhosis

### 2.1. Liver Fibrosis and miRNAs

#### 2.1.1. miRNAs as Biomarkers of Liver Fibrosis

Several miRNAs are associated with organ-specific and systemic fibrosis in the liver [36]. Individual expression of miRNAs in plasma or serum is useful for liver fibrosis detection [3,37]. In addition, some miRNAs can distinguish between early and late fibrosis with high sensitivity and specificity equal to or greater than the APRI and Fib-4 index [38,39]. For example, patients with advanced cirrhosis showed significantly lower levels of miR-29a in their serum compared to healthy controls and patients with early fibrosis [40]. Additionally, serum levels of miR-138 and miR-143 are characteristic of the later stages of liver fibrosis and thus, miR-138 may be useful for detecting fibrosis in its early stages [41]. Furthermore, serum levels of miR-34a and miR-122 correlate with the progression of fibrosis, especially in patients with chronic hepatitis C or nonalcoholic fatty liver disease (NAFLD) [42]. In addition, miR-221 is upregulated in patients with liver cirrhosis [43]. Elucidation of the relationship between miRNAs and liver fibrosis may be useful for detection of fibrosis without invasive liver biopsy, early therapeutic intervention, and identification of high-risk patients. There are various microRNAs that are expected to be biomarkers for liver diseases (Table 1).

#### 2.1.2. miRNAs as Regulators of Liver Fibrosis

Recently, the relationship between liver diseases and various miRNAs has been confirmed (Table 2). Some miRNAs such as miR-21, miR-221/222, and miR-181b, promote liver fibrosis through the TGF-β and NF-κB pathways [77]. In addition, miR-221 regulates multiple targets, including cyclin-dependent kinase inhibitors (CDKN1C or CDKN1B), cytokine signaling 1, E-cadherin, phosphatase and tensin homolog (PTEN), and Bcl-2 modifying factor, which are involved in liver fibrosis [43]. miR-214 also plays an important role in liver fibrosis by regulating the expression of suppressor of fused homolog protein, and knocking down its expression alleviates liver fibrosis in carbon tetrachloride (CCL4)-treated mice [78]. Moreover, the knockdown of the miR-23b miRNA cluster promotes bile duct differentiation and suppresses or restores TGF-β-induced liver fibrosis depending on stellate cell activation [79].

In one study, miR-30a inhibited HSC autophagy, increased lipid accumulation, and improved fibrosis in the livers of mice [80]. miR-29b, miR-101, miR-122, and miR-214-3p prevent fibrosis by inhibiting collagen synthesis and suppressing TGF-β pathway activation [77]. Supplementation with miR-29a improved liver fibrosis in vivo and when administered in advance, it suppressed HSC activation by TGF-β in vitro [81]. miR-29a also plays an important role in the improvement of fibrosis by inhibiting bromodomain-4 protein (BRD4) and the fatty acid translocase protein CD36 [82,83]. Furthermore, overexpression of miR-34 ameliorates the onset and progression of liver fibrosis by regulating the TGF-β1/mothers against decapentaplegic homolog 3 (Smad3) pathway in HSCs [84]. Neutrophils in the liver inhibit liver inflammation and fibrosis by inducing inflammatory macrophages into a reparative phenotype via miR-223 [85]. In addition, miR-455-3p suppresses the expression of heat shock factor 1 and inhibits HSC activation by suppressing the heat shock protein (HSP)-47/TGF-β/Smad4 signaling pathway [86]. Moreover, miR-125b [87], miR-378 [88], and miR-152 [89] can prevent liver fibrosis by regulating the expression of GLI family zinc finger 3 (Gli3).

**Figure 1:**
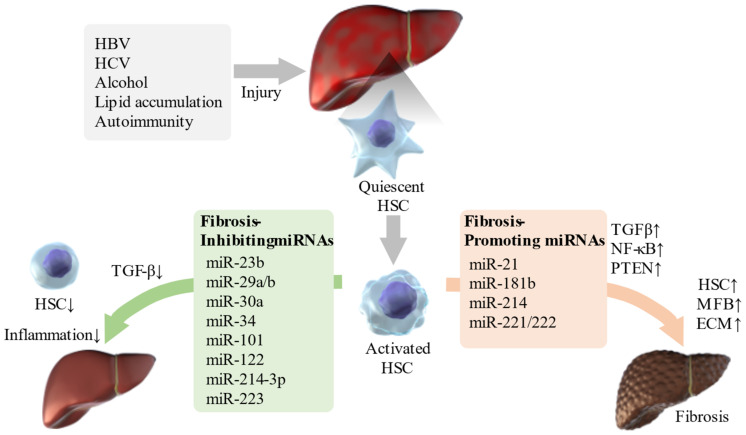
Schematic diagram of the relationship between miRNAs and liver fibrosis. HSCs are activated in livers damaged by viruses, alcohol, lipid accumulation, and autoimmunity. Various miRNAs are involved in this process and affect molecules such as TGF-β, NF-κB, and PTEN, which are involved in different pathways. This can improve inflammation or, conversely, result in ECM accumulation and cirrhosis. HSC: hepatic stellate cell; TGF-β: transforming growth factor-β; NF-κB: nuclear factor-kappa B; PTEN: phosphatase and tensin homolog; MFB: myofibroblast; ECM: extracellular matrix; miRNA: micro ribonucleic acid; HBV: hepatitis B virus; HCV: hepatitis C virus.

Thus, various miRNAs are involved in the regulation of liver fibrosis (Figure 1). The identification of miRNAs involved in the pathogenesis of liver fibrosis will enable miRNA-based therapies, and even if this is difficult, miRNA-associated mechanisms can be targeted for therapy.

### 2.2. Liver Fibrosis and Exosomal miRNAs

HSCs suppress the expression and function of connective tissue growth factor (CTGF also known as CCN2). Of note, miR-214 is upregulated by the transcription factor Twist1; both are highly expressed in quiescent HSCs and in exosomes secreted by HSCs. Moreover, exosome-mediated delivery of miR-214 to HSCs inhibits recipient cell activation via the repression of CCN2 expression. In contrast, Twist and miR-214 are under-expressed in exosomes secreted from activated HSCs. Thus, the Twist-miR-214-CCN2 pathway is one of the mechanisms that regulate HSC activation [113]. Likewise, miR-199a-5p is highly expressed in exosomes secreted from quiescent HSCs and inhibits HSC activation by inhibiting CCN2 activity in the destination HSCs [114].

When hepatocytes are exposed to lipotoxicity, such as excessive fat deposition in the liver tissue, EVs containing miR-128-3p are released. miR-128-3p reaches and is taken up by HSCs, then suppresses peroxisome proliferator-activated receptor (PPAR)-γ function and activates HSCs [115]. In addition, the miR17-92 cluster is highly expressed in the serum exosomes of patients with alcoholic liver disease (ALD), and these miRNAs promote liver fibrosis [116].

Exosomes secreted by fibroblasts contain multiple miRNAs (miR-21, miR-124a, miR-125b, miR-126, miR-130a, and miR-132), HSP-90a, and signal transducer and activator of transcription 3 (STAT3). Fibroblast-derived exosomes enhance the expression of collagen alpha 1 and alpha-smooth muscle actin (alpha-SMA) in tissues and promote fibrosis through the accumulation of ECM. These miRNAs encapsulated in exosomes can promote wound healing and may contribute to tissue fibrosis [117].

Exosomes secreted by mesenchymal stem cells derived from adipose tissue express high levels of miR-122 which is known to have growth- and hepatic fibrosis inhibitory effects on HCC. When these exosomes were administered to CCL4-induced liver injury mice, miR-122 in the exosomes suppressed liver tissue damage and fibrosis via suppression of HSC activation in mice [118].

In contrast to EVs in serum extracted from mice with induced liver fibrosis, miR-34c, miR-151-3p, miR-483-5p, miR-532-5p, and miR-687 were upregulated in serum EVs from mice without fibrosis. When these EVs were administered to mice with CCL4-induced liver injury, hepatocellular damage and liver fibrosis were suppressed in the healthy mouse-derived EV group, and inflammatory cytokines and transaminases in the blood were reduced. Furthermore, in serum EVs from patients with F3/4 hepatic fibrosis and healthy subjects, levels of miR-34c, miR-151-3p, miR-483-5p, and miR-532-5p were upregulated in healthy subjects compared to levels in patients with F3/4 hepatic fibrosis. When EVs from healthy subjects were administered to human-derived HSCs, HSC activation was suppressed. These EV miRNAs suppress HSC activation and contribute to the suppression of liver fibrosis [119].

As described above, there are increasing reports of exosomal miRNAs involved in liver fibrosis, and their clinical application is expected. However, improvement of the quality and accuracy of exosomes and standardization of extraction methods are necessary.

## 3. miRNAs Associated with Complications of Liver Cirrhosis

In addition to HCC, gastrointestinal bleeding due to portal hypertension, ascites, portal vein thrombosis, and HE are some of the most common complications of cirrhosis.

Portal hypertension is caused by an increase in portal venous inflow and intrahepatic vascular resistance, leading to esophageal varices, ascites, HE, and hypersplenism, and it is a major cause of death in patients with cirrhosis [120]. TGF-β-mediated HSC activation is involved in ECM production and is a mechanistic factor in regulating vascular resistance and pressure in the liver [121]. Animal studies have shown that inhibition of TGF-β1 synthesis and blockade of TGF-β receptors can significantly reduce portal hypertensive pressure [122]. Intrahepatic angiogenesis by vascular endothelial growth factor (VEGF), an angiogenic growth factor, is also a cause of sinusoidal systemic circulation and portal hypertension [123]. miR-29 acts as an anti-fibrotic mediator by inhibiting angiogenic factors such as VEGF [90]. The miR-126 family is associated with angiogenesis and directly inhibits negative regulators of the VEGF pathway, such as the Sprouty-related, equine herpesvirus-1 domain-containing protein 1 (SPRED1) and phosphoinositol-3 kinase regulatory subunit 2 (PIK3R2) [91]. Thus, these miRNAs, which affect TGF-β and VEGF, may be key to the treatment of portal hypertension.

A portal vein thrombus in cirrhosis causes worsening of the liver reserve, gastroesophageal varices, and ascites. The pathophysiology of portal vein thrombosis encompasses one or more of the following features: decreased portal blood flow, hypercoagulable state, and damage to the vascular endothelium. As liver function declines, platelets and hepatic-derived coagulation factors are reduced, while hepatic-derived anticoagulation factors are also reduced, and when this balance is disrupted, portal vein thrombi form [124]. Notably, miR-19a and miR-34a levels are correlated with portal vein thrombosis [44], and miR-21 is considered an independent predictor of portal vein thrombosis in patients with HCC [45].

Patients with ascites, spontaneous bacterial peritonitis (SBP), and hepatorenal syndrome had significantly lower levels of miR-122 than those without these complications. In addition, serum miR-122 levels were associated with survival of patients with cirrhosis, independent of the Model for End-Stage Liver Disease (MELD) score or patient’s age [46]. One study indicated that miR-155 is an outstanding diagnostic marker for SBP, and detection of both serum CD64 and calprotectin levels also provide a more useful diagnosis when using blood samples from patients with cirrhosis and ascites [47]. Furthermore, miR-155 [48] and miR-223 [49] were elevated in ascites from patients with cirrhosis and SBP, indicating that these miRNAs may be involved in the immune response in ascites upon SBP. Animal models of HE also show changes in miRNAs in the blood and cerebral cortex [125,126]. In the HE model, ammonia-induced changes in miRNA expression regulate the expression of heme oxygenase 1 (HO-1) and induce astrocyte senescence [127].

Complications of cirrhosis often occur in the non-compensated phase, so there is little time to wait for the improvement of liver fibrosis, and a direct approach is needed for diagnosis and treatment. Therefore, miRNA-based diagnostic and therapeutic approaches should be developed.

## 4. Association of the Causes of Liver Cirrhosis and miRNAs

### 4.1. Chronic Hepatitis B Virus (HBV) Infection and miRNAs

An HBV infection causes acute and chronic hepatitis, cirrhosis, and HCC. The annual incidence of cirrhosis from a chronic HBV infection was 2.1~6.0% [128,129]. Although antiviral treatment with peginterferon-alpha or nucleic acid analogs inhibits fibrosis progression [130,131], there is still no treatment to improve HBV-related cirrhosis once treatment is completed.

Toll-like receptors (TLRs) and several miRNAs involved in the TLR signaling pathway play important roles in innate immunity against HBV infection [132]. miR-21 [50], miR-22 [133,134], miR-122 [133,134,135,136,137], miR-194 [137], and miR-219-1 [138] are associated with chronic persistent HBV infections. During the progression from chronic hepatitis to cirrhosis and HCC, miR-21, miR-199b, miR-145, and miR-602 were aberrantly expressed from the initial stage to the end stage [50]. In HBV infections, miR-22 is involved in the regulation of cell fate and development of HCC [133,134]. Overexpression of miR-122 downregulates HO-1 and inhibits HBV expression [139]. miR-122 and miR-22 are downregulated in patients with HBV-associated HCC, are associated with HCC development and progression, and correlate with clinical and pathological indicators [140]. Additionally, miR-219-1 is associated with the clearance of HBV infections and may influence the outcome of persistent HBV infections [138].

Liver biopsies of HBV-infected patients indicate a correlation between miRNAs and liver fibrosis [141,142]. In early liver fibrosis, miR-34b-3p, miR-1224-3p, and miR-1227-3p expression is increased, and miR-499a-5p expression is decreased. Contrastingly, in advanced hepatic fibrosis, miR-1, miR-10b-5p, miR-96-5p, miR-133b, and miR-671-5p are upregulated, while miR-20b-5p and miR-455-3p are downregulated [141,142]. Moreover, miR-21-5p expression is strongly positively correlated with hepatic fibrosis and causes HBV-induced hepatic fibrosis via TGF-β1 signaling [50]. miR-125a-5p levels are significantly increased in patients with cirrhosis, and miR-125a-5p may be a novel biomarker for liver injury [51]. miR-27a is elevated in HBV-associated cirrhosis and is a predictor of HSC activation, differentiation, and proliferation [52]. miR-181b activates HSCs through the PTEN/protein kinase B (Akt) pathway and has been identified as an independent predictor of disease progression in HBV [53].

Although hepatitis B viral load can be controlled by nucleic acid analogs and interferons, carcinogenesis cannot be completely prevented. Elucidating the relationship between HBV and miRNAs is expected to lead to breakthroughs in the treatment of HBV.

### 4.2. Chronic Hepatitis C Virus (HCV) Infection and miRNAs

HCV infections are one of the most serious health problems worldwide. More than 170 million people are chronically infected with HCV and are at a high risk of developing liver cirrhosis and HCC. HCV infections cause acute and chronic hepatitis, cirrhosis, and HCC. The annual incidence of cirrhosis due to hepatitis C is 1.1% [129]. Although the development of treatment for hepatitis C has made it possible to eradicate the disease, it is unclear to what extent fibrosis will improve in patients who would have already progressed to cirrhosis. In addition, there is no 100% inhibition of carcinogenesis. Several miRNAs are associated with liver fibrosis in HCV infections.

Serum miRNAs are considered important non-invasive biomarkers of advanced stages of HCV-related liver fibrosis [143]. miR-16, miR-146a, miR-221, and miR-222 are upregulated in early and late fibrosis, and miR-222 and miR-221 exhibit high sensitivity and specificity in late fibrosis [54]. Liver biopsies of HCV-related liver fibrosis samples indicate upregulation of miR-21 [56], and this miRNA enhances TGF-β signaling by targeting SMAD7, a negative regulator of TGF-β, and consequently induces fibrogenesis [56]. miR-16, miR-34a, and miR-221 are elevated in liver damage caused by HCV and can be used to detect fibrosis and cirrhosis [55]. Compared with patients with mild fibrosis, five circulating miRNAs (miR-215-5p, miR-483-5p, miR-193b-3p, miR-34a-5p, and miR-885-5p) showed increased expression, and two miRNAs (miR-26b-5p and miR-197-3p) showed decreased expression in patients with HCV cirrhosis [58]. miR-122 and miR-130a play important roles in chronic hepatitis C [132]. miR-122 stimulates HCV translation, stabilizes the genome, and induces viral genomic RNA replication [144]. Additionally, miR-122 is negatively correlated with fibrosis in HCV-infected patients [59]. The expression of miR-20a is significantly upregulated in the sera of patients with HCV-associated liver fibrosis and gradually increased from the early to late stages of fibrosis [61].

The expression of miR-99a was significantly lower in patients with chronic HCV infection than in healthy subjects. miR-99a modulates the expression of the mammalian target of rapamycin protein (mTOR) to improve intracellular lipid accumulation and limit HCV replication [57]. miR-200c, which is increased in HCV-infected patients, regulates the Src kinase signaling pathway and promotes liver fibrosis by directly targeting Fas-associated phosphatase 1 (FAP-1), a negative regulator of Src signaling [60]. 

Direct-acting antivirals are highly effective, but HCV elimination is not a cure for liver disease, especially in patients with advanced fibrosis or cirrhosis. However, miRNAs show promise as a treatment for residual liver fibrosis after antiviral treatment.

### 4.3. ALD and miRNAs

ALD is the leading cause of chronic liver disease, liver fibrosis, and cirrhosis worldwide [145], and Kupffer cells play an important role in the development of ALD. The human intestinal tract contains many bacteria which produce endotoxins. The gut-derived endotoxins are transported to the liver, where they are removed by Kupffer cells. Studies have shown that these endotoxins activate Kupffer cells. Two mechanisms have been suggested to explain the relationship between endotoxin levels and alcohol intake: (i) chronic alcohol consumption increases endotoxin levels in the circulating blood because Kupffer cells are unable to effectively remove endotoxins from the blood, and (ii) alcohol consumption increases intestinal permeability, leading to increased intestinal endotoxin absorption [145].

Serum miR-122, miR-223, miR-155, and miR-146a levels are increased in ALD [146,147]. miR-122 protects the liver from inflammation by decreasing the expression of hypoxia-inducible factor 1α(HIF-1α) in the liver [92]. Previous studies show that the levels of neutrophil-specific miR-233 in the serum and liver were elevated in both patients with ALD and animal models. This miRNA plays an important role in inhibiting neutrophil hyperactivation by targeting the IL-6-p47phox pathway in neutrophils [93]. miR-155, a major regulator of inflammation and immunity, promotes inflammation in alcoholic steatohepatitis (ASH). Alcohol induces miR-155 expression in the liver via the TLR4 pathway, and miR-155 promotes liver fibrosis by targeting peroxisome proliferator response elements (PPREs) and PPAR-α [94]. Ethanol regulates innate immune activity and causes ethanol-induced liver injury. Of note, miR-181b-3p suppresses inflammation via targeting importin α5 and normalizes lipopolysaccharide-stimulated TNFα expression in Kupffer cells [95]. miR-217 promotes fat accumulation in hepatocytes in ALD by suppressing sirtuin 1. In addition, it reduces the function of lipin-1, an important regulator of lipids in hepatocytes [96].

Although ALD is a global problem, there is limited progress its treatment. The lack of therapeutic progress in the field of ALD is partly due to the lack of experimental models of advanced ALD and the difficulty of conducting clinical trials in active addicts. However, miRNA research may lead to a breakthrough in ALD treatment.

### 4.4. NASH/NAFLD and miRNAs

NAFLD is one of the most common causes of chronic liver disease and is a serious medical problem in developed countries [148]. In patients with NAFLD, the degree of liver damage varies from NAFL to NASH and may progress to HCC in the early stages of liver fibrosis [149]. NASH is mediated by inflammatory cytokines, mitochondrial dysfunction secondary to nutrient excess, and oxidative stress, resulting in hepatocyte inflammation, ballooning, apoptosis, and activation of HSCs [150,151]. Numerous factors have been implicated in the pathogenesis and progression of NAFLD, including insulin resistance, adipose tissue dysfunction, mitochondrial dysfunction, endoplasmic reticulum stress, dietary factors, fatty acids, iron loading, inflammatory activation, lipopolysaccharide produced by the gut microbiota, chronic inflammatory conditions, and the involvement of genetic and epigenetic factors [152,153]. However, the detailed mechanisms of NAFLD/NASH remain unknown, and there is no breakthrough treatment.

There have been many studies on the relationship between pathogenesis and miRNAs in NASH/NAFLD [154]. Serum miRNA-34a is effective in diagnosing NAFLD. In lipid metabolism, it may downregulate the PPARα signaling pathway and induce lipid accumulation in hepatocytes [63]. Neutrophil-specific miR-223 is upregulated in hepatocytes and inhibits the progression of NASH in obese mice. Some of the target genes of miR-223 (such as *CXCL10*, *NLRP3*, and *TAZ*) induce inflammation and fibrosis in the liver and promote the progression of NAFLD. EV-derived miR-223, when taken up by hepatocytes, suppresses hepatic inflammatory and fibrogenic gene expression [102]. miR-372-3p and miR-373-3p, which downregulate adipocyte enhancer binding protein 1 (AEBP1), are reduced in patients with NASH and advanced fibrosis [64]. 

miR-21 induces NASH via the STAT3 signaling pathway and induces liver fibrosis via HSC activation and collagen deposition via the TGF-β/Smad3/Smad7 signaling pathway [97]. Overexpression of hepatic miR-27 promotes the expression of hepatic insulin receptors, while inhibition decreases insulin sensitivity, suggesting that miR-27 may contribute to the early development of hepatic insulin resistance [103]. In one study, the overexpression of miR-29a ameliorated NASH and NAFLD by suppressing CD36 in a mouse model [98]. Another study demonstrated that decreasing miR-122 increased fat deposition and total triglyceride content in the liver and decreased beta-oxidation and energy expenditure, resulting in increased weight gain in mice [99]. miR-34a downregulates the PPARα signaling pathway, which is a key transcription factor for fatty acid oxidation and facilitates the transfer of fatty acids to mitochondria for oxidation. Conversely, blocking the PPARα signaling pathway may induce lipid accumulation in the liver [62]. miR-129-5p negatively regulates HSC activation induced by paternally expressed gene 3 (PEG3) [100]. Inhibition of miR-188-5p alleviates liver fibrosis by suppressing HSC activation through the PTEN/PI3K/Akt pathway [101].

Since the incidence of NAFLD/NASH is expected to continue to increase, there is an urgent need to develop early diagnosis and treatment methods using miRNAs.

### 4.5. Autoimmune Liver Diseases and miRNAs

Autoimmune liver diseases include autoimmune hepatitis (AIH), primary biliary cholangitis (PBC), and primary sclerosing cholangitis (PSC), which are characterized by chronic liver and biliary inflammation. These diseases require persistent treatment, but the underlying causes of these diseases are still unknown. To date, numerous susceptible loci for autoimmune liver diseases in the human leukocyte antigen (HLA) and non-HLA regions have been identified by a genome-wide association studies (GWAS) [155,156]. However, genetic analysis alone is inadequate to identify the cause of autoimmune liver diseases, and environmental factors may be involved in the development of these diseases.

In recent years, the pathological and diagnostic relevance of miRNAs in autoimmune liver diseases have been reported.

#### 4.5.1. AIH and miRNAs

AIH is a chronic, progressive liver disease that usually occurs in middle-aged and older women, and autoimmune mechanisms have been implicated in the development of this hepatocellular damage [157,158]. In patients with untreated AIH, serum levels of miR-122 and miR-21 are significantly elevated but decrease during remission with glucocorticoid treatment [67]. In addition, miR-122 and miR-21 levels are negatively correlated with liver fibrosis [67].

Using a concanavalin A (Con A)-induced AIH mouse model, miRNA expression was altered in the mice compared to a control group. Moreover, expression of miR-133a was increased in the AIH mouse model [65,66]. After Con A treatment, the levels of miR-375, IL-6, IL-1β, and TNF-α were increased in Kupffer cells. Furthermore, a miR-375 inhibitor decreased apoptosis in Kupffer cells by targeting astrocyte elevated gene 1 (AEG1) and restoring immune abnormalities in the liver [104]. Downregulation of miR-138 affected liver macrophage function and improved immune status by targeting p53 in an AIH mouse model [105]. Reduction of miR-15a/16-1 in damaged hepatocytes contributes to IL-22-mediated tissue repair by decreasing cell apoptosis and promoting cell proliferation [106]. In Con A-induced murine immune hepatotoxicity, 5-lipoxygenase (5-LO) is involved in the production of cysteinyl-leukotrienes which exacerbate hepatotoxicity. miR-674-5p may ameliorate liver injury by negatively regulating the expression of 5-LO [107]. miRNA-143 regulates inflammation and fibrosis by regulating the phosphorylation of TGF-β-activated kinase 1 (TAK1) [108]. miRNAs modulate apoptosis and inflammatory reactions by affecting macrophages, regulatory T cells, Th17 cells, CD4^+^ T cells, and hepatocytes [67]. For example, miR-223 suppressed Kupffer cell activation in a mouse model of AIH by decreasing IL-1β secretion via the absent in melanoma 2 (AIM2) pathway [109].

Since AIH is a disease with an unknown cause, this delays diagnosis, which can be fatal, and there is an urgent need to develop new diagnostic and therapeutic methods using miRNAs and other technologies.

#### 4.5.2. PBC and miRNAs

PBC is a progressive cholestatic liver disease that is caused by a combination of genetic predisposition and environmental triggers [157]. It is characterized by chronic non-suppurative inflammation and destruction of interlobular bile ducts, which in turn may lead to liver cirrhosis, liver failure, and death, unless liver transplantation is undertaken. miR-21 and miR-210 levels in the liver tissue of patients with PBC are increased compared to those in healthy individuals [68,69]. Elevation of a let-7 miRNA (let-7b) and miR-520a-5p, and a decrease in miR-125b, are biomarkers in refractory PBC [70]. The expression of miR-223-3p and miR-21-5p was suppressed in the peripheral blood B cells of patients with PBC as the PBC stage progressed [72]. miR-139-5p targets the proto-oncogene, c-FOS, and promotes the NF-κB signaling pathway, leading to an increase in TNF-α levels [73]. It was significantly downregulated in clinically advanced PBC (portal hypertension type and hepatic failure type). Increased levels of immunomodulatory miR-451a and miR-642a-3p were observed in plasma-derived EVs of patients with PBC compared to the levels in healthy controls [159].

miRNAs regulate immune responses, hepatocyte apoptosis, bile acid metabolism, and biliary fibrosis by inhibiting various signaling pathways in PBC. miR-21 targets cyclin-dependent kinase 2-associated protein 1 (CDK2AP1), and inhibition of miR-21 ameliorates liver damage, necrosis, and fibrosis [68,69]. miR-506 plays an important role in the pathogenesis of PBC by regulating bicarbonate secretion [67]. In addition, miR-92a was downregulated in patients with PBC, suggesting that it directly regulates IL-17A and is involved in disease progression [71].

The diagnosis of PBC is relatively straightforward if the anti-mitochondrial antibody (AMA) test is positive. However, there are patients who may have negative AMA test results, and these patients require a liver biopsy for diagnosis. This can be problematic in the presence of ascites or an infection, and noninvasive biomarkers are needed. In addition, PBC is gradually progressive, and some patients are refractory to treatment and may not recover. Consequently, miRNAs may be useful in the diagnosis and treatment of such cases.

#### 4.5.3. PSC and miRNAs

In PSC, T cell-related risk genes have a strong correlation with disease progression. Dysfunctional gene products can disrupt immune homeostasis by regulating cytokine production, immune tolerance, and immune responses [157]. Unfortunately, there are no effective drugs to slow down the natural progression of PSC, and liver transplantation is the only option for advanced PSC. Three miRNAs (miR-3178, miR-4484, and miR-150-5p) were abnormally altered in serum samples [74]. Low miRNA-122 levels in the serum may be associated with a poor prognosis [75]. The downregulation of miR-200c may be a new diagnostic biomarker for PSC detection [76].

miRNAs are involved in the pathogenesis of PSC through regulation of angiogenesis, apoptosis, cholangiocyte proliferation, and liver fibrosis. In isolated cholangiocytes from a model of sclerosing cholangitis created by treatment with 3,5-diethoxycarbonyl-1,4-dihydrocollidine (DDC), the expression of miR-7a and the transcription factor neurogenin-3 was increased, resulting in the proliferation of cholangiocytes [110]. miR-873-5p targets glycine N-methyltransferase (GNMT). A deficiency of GNMT exacerbates fibrogenesis caused by bile stasis causing high blood levels of miR-873-5p in patients with bile stasis and liver cirrhosis. In addition, administration of an anti-miR-873-5p antibody to a PSC mouse model restored GNMT levels and ameliorated inflammation and fibrosis [111]. Inhibition of miR-24 increases menin and TGF-β1 expression and exacerbates liver fibrosis in a mouse model of PSC [112].

The only treatment for advanced PSC is liver transplantation, and early diagnosis and treatment using miRNAs might be established in the future.

## 5. Conclusions

Despite the excellent disease control by antiviral therapy for viral liver diseases, no treatment has yet been established to improve liver fibrosis once it has progressed. In addition, there is an urgent need to establish biomarkers for the diagnosis of liver fibrosis that do not require invasive liver biopsy.

Some liver miRNAs have been reported to be associated with hepatic fibrosis, and the establishment of these miRNAs may lead to early detection and better treatment for liver cirrhosis. In fact, accumulating evidence has revealed that miRNAs (including exosomal miRNAs) play important roles in many biological processes involved in liver fibrosis, including viral hepatitis, ALD, NASH, and autoimmune liver diseases. Several studies have demonstrated the relationship between various pathogens and miRNAs as biomarkers for liver fibrosis. Furthermore, elucidating the details of miRNAs involved in liver fibrosis and the mechanisms of their regulation will be beneficial for the development of anti-fibrosis therapies. A combination of conventional and miRNA-based therapies may be effective in the treatment of advanced liver cirrhosis. However, the regulation of miRNAs in vivo may not always be observed due to their inherent complexity, and further studies to elucidate their detailed mechanisms are required for the clinical application of miRNAs. Future studies of miRNAs in liver fibrosis will bring about the usefulness of miRNAs in clinical applications for the treatment of liver cirrhosis.

## Figures and Tables

**Table 1:** miRNA as biomarker of liver fibrosis.

	miRNA	Expression Level	References
Liver fibrosis	miR-29a	Down	[40]
Liver fibrosis	miR-138	Up	[41]
Liver fibrosis	miR-143	Up	[41]
Liver fibrosis	miR-34a	Up	[42]
Liver fibrosis	miR-122	Up	[42]
Liver fibrosis	miR-221	Up	[43]
Portal vein thrombus	miR-19a	Up	[44]
Portal vein thrombus	miR-34a	Up	[44]
Portal vein thrombus	miR-21	Up	[45]
SBP	miR-122	Down	[46]
SBP	miR-155	Up	[47,48]
SBP	miR-223	Up	[49]
HBV	miR-21-5p	Up	[50]
HBV	miR-125a-5p	Up	[51]
HBV	miR-27a	Up	[52]
HBV	miR-181b	Up	[53]
HCV	miR-222	Up	[54]
HCV	miR-221	Up	[54,55]
HCV	miR-21	Up	[56]
HCV	miR-99a	Down	[57]
HCV	miR-215-5p	Up	[58]
HCV	miR-483-5p	Up	[58]
HCV	miR-193b-3p	Up	[58]
HCV	miR-34a	Up	[55,58]
HCV	miR-885-5p	Up	[58]
HCV	miR-26b-5p	Down	[58]
HCV	miR-197-3p	Down	[58]
HCV	miR-122	Down	[59]
HCV	miR-16	Up	[55]
HCV	miR-200c	Up	[60]
HCV	miR-20a	Up	[61]
NASH/NAFLD	miR-34a	Up	[62,63]
NASH/NAFLD	miR-372-3p	Down	[64]
NASH/NAFLD	miR-373-3p	Down	[64]
AIH	miR-133a	Up	[65,66]
AIH	miR-122	Down	[67]
AIH	miR-21	Down	[67]
PBC	miR-21	Up	[68]
PBC	miR-210	Up	[69]
PBC	let-7b	Up	[70]
PBC	miR-520a-5p	Up	[70]
PBC	miR-125b	Down	[70]
PBC	miR-92a	Down	[71]
PBC	miR-223-3p	Down	[72]
PBC	miR-21-5p	Down	[72]
PBC	miR-139-5p	Down	[73]
PSC	miR-150-5p	Down	[74]
PSC	miR-122	Down	[75]
PSC	miR-200c	Down	[76]

**Table 2:** Fibrosis-associated miRNAs.

	miRNA	Predicted Target	Involvement in Disease Progression	References
Liver fibrosis	miR-21	TGF-β pathway, NF-κB pathway	promote	[77]
Liver fibrosis	miR-221/222	TGF-β pathway, NF-κB pathway	promote	[77]
Liver fibrosis	miR-181b	TGF-β pathway, NF-κB pathway	promote	[77]
Liver fibrosis	miR-221	CDKN1C, CDKN1B, Socs1, E-cadherin, PTEN, BMF	promote	[43]
Liver fibrosis	miR-214	Sufu	promote	[78]
Liver fibrosis	miR-23b	TGF-β pathway	inhibit	[79]
Liver fibrosis	miR-30a	Beclin1,α-SMA,TIMP-1,Collagen I	inhibit	[80]
Liver fibrosis	miR-29b	TGF-β pathway	inhibit	[77]
Liver fibrosis	miR-101	TGF-β pathway	inhibit	[77]
Liver fibrosis	miR-122	TGF-β pathway	inhibit	[77]
Liver fibrosis	miR-214-3p	TGF-β pathway	inhibit	[77]
Liver fibrosis	miR-29a	BRD4, CD36	inhibit	[81,82,83]
Liver fibrosis	miR-34	TGF-β1/Smad3 pathway	inhibit	[84]
Liver fibrosis	miR-223	NLRP3	inhibit	[85]
Liver fibrosis	miR-455-3p	HSF1	inhibit	[86]
Liver fibrosis	miR-125b	Gli3	inhibit	[87]
Liver fibrosis	miR-378	Gli3	inhibit	[88]
Liver fibrosis	miR-152	Gli3	inhibit	[89]
Portal hypertension	miR-29	VEGF	inhibit	[90]
Portal hypertension	miR-126 family	SPRED1, PIK3R2/p85-beta	inhibit	[91]
HBV	miR-21-5p	TGF-β1	promote	[50]
HBV	miR-27a	PPARγ, FOXO1, APC, P53, RXRα	promote	[52]
HBV	miR-181b	PTEN/Akt pathway	promote	[53]
HCV	miR-21	Smad7	promote	[56]
HCV	miR-99a	mTOR	inhibit	[57]
HCV	miR-200c	FAP-1	promote	[60]
ALD	miR-122	HIF1α	inhibit	[92]
ALD	miR-223	IL-6-p47phox pathway	inhibit	[93]
ALD	miR-155	PPARγ, PPRE	promote	[94]
ALD	miR181b-3p	importin α5	inhibit	[95]
ALD	miR-217	SIRT1	promote	[96]
NASH/NAFLD	miR-21	STAT3 signaling pathway, TGF-β/Smad3/Smad7 signaling pathway	promote	[97]
NASH/NAFLD	miR-29a	CD36	inhibit	[98]
NASH/NAFLD	miR-122	AGPAT1, DGAT1	inhibit	[99]
NASH/NAFLD	miR-34a	PPARα signaling pathway	promote	[62,63]
NASH/NAFLD	miR-129-5p	PEG3	inhibit	[100]
NASH/NAFLD	miR-188-5p	PTEN/PI3K/AKT pathway	promote	[101]
NASH/NAFLD	miR-223	Cxcl10, Nlrp3, Taz	inhibit	[102]
NASH/NAFLD	miR-27	insulin signaling pathway	promote	[103]
NASH/NAFLD	miR-372-3p	AEBP1	inhibit	[64]
NASH/NAFLD	miR-373-3p	AEBP1	inhibit	[64]
AIH	miR-375	AEG-1	promote	[104]
AIH	miR-138	p53	promote	[105]
AIH	miR-15a/miR-16-1	aryl hydrocarbon receptor-IL-22 regulatory axis	promote	[106]
AIH	miR-674-5p	5-LO	inhibit	[107]
AIH	miRNA-143	phosphorylation of TAK1	inhibit	[108]
AIH	miR-223	AIM2	inhibit	[109]
PBC	miR-21	CDK2AP1	promote	[68]
PBC	miR-210	MLL4	promote	[69]
PBC	miR-506	AE 2, InsP3R3	promote	[67]
PBC	miR-92a	IL-17A	inhibit	[71]
PBC	miR-223-3p	TGFBR2,MEF2C,FOXP1,RBPJ	promote	[72]
PBC	miR-21-5p	TGFBR2,MEF2C,FOXP2,RBPJ	promote	[50]
PBC	miR-139-5p	c-FOS	promote	[73]
PSC	miR-7a	Ngn-3	promote	[110]
PSC	miR-873-5p	GNMT	promote	[111]
PSC	miR-24	menin	inhibit	[112]

## Abbreviations

5-LO	5-lipoxygenase
AE 2	Anion exchanger 2
AEBP1	Adipocyte enhancer binding protein 1
AEG-1	Astrocyte elevated gene 1
AGPAT1	1-acylglycerol-3-phosphate O-acyltransferase 1
AIH	Autoimmune hepatitis
AIM2	Absent in melanoma 2
ALD	Alcoholic liver disease
alpha-SMA	Alpha-smooth muscle actin
AMA	Anti-mitochondrial antibody
APC	Adenomatous polyposis coli
ASH	Alcoholic steatohepatitis
BMF	Bcl-2 modifying factor
BRD4	Bromodomain-4 protein
CCL4	Carbon tetrachloride
CDK2AP1	Cyclin dependent kinase 2 associated protein 1
CDKN	Cyclin-dependent kinase inhibitor
ConA	Concanavalin A
CTGF	Connective tissue growth factor
CXCL10	C-X-C motif chemokine ligand 10
cys-LT	Cysteinyl-leukotrienes
DDC	3,5-diethoxycarbonyl-1,4-dihydrocollidine
DGAT1	Diacylglycerol O-acyltransferase 1
ECM	Extracellular matrix
EV	Extracellular vesicle
FAP-1	FAS-associated phosphatase 1
FOXO1	Forkhead box protein O1
FOXP1	Forkhead box P1
Gli3	GLI family zinc finger 3
GNMT	Glycine N-methyltransferase
GWAS	Genome-wide association study
HBV	Hepatitis B virus
HCC	Hepatocellular carcinoma
HCV	Hepatitis C virus
HE	Hepatic encephalopathy
HIF1α	Hypoxia inducible factor α
HLA	Human leukocyte antigen
HO-1	Heme oxygenase 1
HSC	Hepatic stellate cell
HSF1	Heat shock factor 1
HSP	Heat shock protein
IL	Interleukin
InsP3R3	Type III inositol 1,4,5-trisphosphate receptor-3
LPS	Lipopolysaccharide
M2BPGi	Mac-2-binding protein glycosylation isomer
MEF2C	Myocyte enhancer factor 2C
MFB	Myofibroblast
miRNA	MicroRNA
MLL4	Histone methyltransferase mixed-lineage leukemia-4
mRNA	Messenger RNA
mTOR	Mammalian target of rapamycin protein
NAFLD	Nonalcoholic fatty liver disease
NASH	Nonalcoholic steatohepatitis
ncRNA	Non-coding RNA
NF-κB	Nuclear factor-kappa B
NKT	Natural killer T
NLRP3	NLR family pyrin domain containing 3
PBC	Primary biliary cholangitis
PDGF	Platelet-derived growth factor
PEG3	Paternally expressed gene 3
PIK3R2	Phosphoinositol-3 kinase regulatory subunit 2
PPAR	Peroxisome proliferator-activated receptor
PSC	Primary sclerosing cholangitis
PTEN	Phosphatase and tensin homolog
RBPJ	Recombination signal binding protein for immunoglobulin kappa J region
RXRα	Retinoid X receptor alpha
SBP	Spontaneous bacterial peritonitis
SIRT1	Sirtuin 1
Smad	Mothers against decapentaplegic homolog
Socs1	Suppressor of cytokine signaling 1
SPRED1	Sprouty related EVH1 domain containing 1
STAT3	Signal transducer and activator of transcription 3
TAK1	TGF-beta activated kinase 1
TAZ	Tafazzin
TGF	Transforming growth factor
TGFBR2	Transforming growth factor beta receptor 2
TLR	Toll-like receptor
TNF	Tumor necrosis factor
VEGF	Vascular endothelial growth factor

## References

[1] Paul S., Ruiz-Manriquez L.M., Serrano-Cano F.I., Estrada-Meza C., Solorio-Diaz K.A., Srivastava A. (2020). Human microRNAs in host-parasite interaction: A review. 3 Biotech.

[2] Castera L. (2012). Noninvasive methods to assess liver disease in patients with hepatitis B or C. Gastroenterology.

[3] Iacob D.G., Rosca A., Ruta S.M. (2020). Circulating microRNAs as non-invasive biomarkers for hepatitis B virus liver fibrosis. World J. Gastroenterol..

[4] Ogawa M., Tsuchiya A., Watanabe T., Setsu T., Kimura N., Matsuda M., Hoshiyama Y., Saito H., Kanazawa T., Shiotani M. (2020). Screening and follow-up of chronic liver diseases with understanding their etiology in clinics and hospitals. JGH Open.

[5] Koyama Y., Brenner D.A. (2017). Liver inflammation and fibrosis. J. Clin. Investig..

[6] Seki E., Brenner D.A. (2015). Recent advancement of molecular mechanisms of liver fibrosis. J. Hepato Biliary Pancreat. Sci..

[7] Higashi T., Friedman S.L., Hoshida Y. (2017). Hepatic stellate cells as key target in liver fibrosis. Adv. Drug Deliv. Rev..

[8] Lau N.C., Lim L.P., Weinstein E.G., Bartel D.P. (2001). An abundant class of tiny RNAs with probable regulatory roles in *Caenorhabditis elegans*. Science.

[9] Lagos-Quintana M., Rauhut R., Lendeckel W., Tuschl T. (2001). Identification of novel genes coding for small expressed RNAs. Science.

[10] Lambrecht J., Verhulst S., Mannaerts I., Reynaert H., van Grunsven L.A. (2018). Prospects in non-invasive assessment of liver fibrosis: Liquid biopsy as the future gold standard?. Biochim. Biophys. Acta Mol. Basis Dis..

[11] O’Brien K., Breyne K., Ughetto S., Laurent L.C., Breakefield X.O. (2020). RNA delivery by extracellular vesicles in mammalian cells and its applications. Nat. Rev. Mol. Cell Biol..

[12] Kogure A., Kosaka N., Ochiya T. (2019). Cross-talk between cancer cells and their neighbors via miRNA in extracellular vesicles: An emerging player in cancer metastasis. J. Biomed. Sci..

[13] Forouzanfar M.H., Alexander L., Anderson H.R., Bachman V.F., Biryukov S., Brauer M., Burnett R., Casey D., Coates M.M., GBD 2013 Risk Factors Collaborators (2015). Global, regional, and national comparative risk assessment of 79 behavioural, environmental and occupational, and metabolic risks or clusters of risks in 188 countries, 1990–2013: A systematic analysis for the Global Burden of Disease Study 2013. Lancet.

[14] Tsochatzis E.A., Bosch J., Burroughs A.K. (2014). Liver cirrhosis. Lancet.

[15] Zhou W.C., Zhang Q.B., Qiao L. (2014). Pathogenesis of liver cirrhosis. World J. Gastroenterol..

[16] Blomhoff R., Wake K. (1991). Perisinusoidal stellate cells of the liver: Important roles in retinol metabolism and fibrosis. FASEB J..

[17] Winau F., Hegasy G., Weiskirchen R., Weber S., Cassan C., Sieling P.A., Modlin R.L., Liblau R.S., Gressner A.M., Kaufmann S.H. (2007). Ito cells are liver-resident antigen-presenting cells for activating T cell responses. Immunity.

[18] Kaps L., Schuppan D. (2020). Targeting cancer associated fibroblasts in liver fibrosis and liver cancer using nanocarriers. Cells.

[19] Parola M., Pinzani M. (2019). Liver fibrosis: Pathophysiology, pathogenetic targets and clinical issues. Mol. Asp. Med..

[20] Tsuchida T., Friedman S.L. (2017). Mechanisms of hepatic stellate cell activation. Nat. Rev. Gastroenterol. Hepatol..

[21] Takahashi K., Yan I., Haga H., Patel T. (2014). Long noncoding RNA in liver diseases. Hepatology.

[22] Friedman R.C., Farh K.K., Burge C.B., Bartel D.P. (2009). Most mammalian mRNAs are conserved targets of microRNAs. Genome Res..

[23] O’Brien J., Hayder H., Zayed Y., Peng C. (2018). Overview of microRNA biogenesis, mechanisms of actions, and circulation. Front. Endocrinol..

[24] McGeary S.E., Lin K.S., Shi C.Y., Pham T.M., Bisaria N., Kelley G.M., Bartel D.P. (2019). The biochemical basis of microRNA targeting efficacy. Science.

[25] Takahashi K., Yan I., Wen H.J., Patel T. (2013). MicroRNAs in liver disease: From diagnostics to therapeutics. Clin. Biochem..

[26] Oura K., Morishita A., Masaki T. (2020). Molecular and functional roles of microRNAs in the progression of hepatocellular carcinoma—A review. Int. J. Mol. Sci..

[27] Morishita A., Oura K., Tadokoro T., Fujita K., Tani J., Masaki T. (2021). MicroRNAs in the pathogenesis of hepatocellular carcinoma: A review. Cancers.

[28] Morishita A., Oura K., Tadokoro T., Fujita K., Tani J., Masaki T. (2021). MicroRNA interference in hepatic host-pathogen interactions. Int. J. Mol. Sci..

[29] Morishita A., Fujita K., Iwama H., Chiyo T., Fujihara S., Oura K., Tadokoro T., Mimura S., Nomura T., Tani J. (2020). Role of microRNA-210-3p in hepatitis B virus-related hepatocellular carcinoma. Am. J. Physiol. Gastrointest. Liver Physiol..

[30] Morishita A., Masaki T. (2018). MicroRNAs as possible biomarkers for hepatocellular carcinoma. Hepatol. Res..

[31] Miyata M., Morishita A., Sakamoto T., Katsura A., Kato K., Nishioka T., Toyota Y., Fujita K., Maeda E., Nomura T. (2015). MicroRNA profiles in cisplatin-induced apoptosis of hepatocellular carcinoma cells. Int. J. Oncol..

[32] Oura K., Tadokoro T., Fujihara S., Morishita A., Chiyo T., Samukawa E., Yamana Y., Fujita K., Sakamoto T., Nomura T. (2017). Telmisartan inhibits hepatocellular carcinoma cell proliferation In Vitro by inducing cell cycle arrest. Oncol. Rep..

[33] Valadi H., Ekstrom K., Bossios A., Sjostrand M., Lee J.J., Lotvall J.O. (2007). Exosome-mediated transfer of mRNAs and microRNAs is a novel mechanism of genetic exchange between cells. Nat. Cell Biol..

[34] Kalluri R., LeBleu V.S. (2020). The biology, function, and biomedical applications of exosomes. Science.

[35] Yamayoshi A., Oyama S., Kishimoto Y., Konishi R., Yamamoto T., Kobori A., Harada H., Ashihara E., Sugiyama H., Murakami A. (2020). Development of antibody-oligonucleotide complexes for targeting exosomal microRNA. Pharmaceutics.

[36] O’Reilly S. (2016). MicroRNAs in fibrosis: Opportunities and challenges. Arthritis Res. Ther..

[37] Wang J., Chu E.S., Chen H.Y., Man K., Go M.Y., Huang X.R., Lan H.Y., Sung J.J., Yu J. (2015). MicroRNA-29b prevents liver fibrosis by attenuating hepatic stellate cell activation and inducing apoptosis through targeting PI3K/AKT pathway. Oncotarget.

[38] Wang T.Z., Lin D.D., Jin B.X., Sun X.Y., Li N. (2019). Plasma microRNA: A novel non-invasive biomarker for HBV-associated liver fibrosis staging. Exp. Ther. Med..

[39] Appourchaux K., Dokmak S., Resche-Rigon M., Treton X., Lapalus M., Gattolliat C.H., Porchet E., Martinot-Peignoux M., Boyer N., Vidaud M. (2016). MicroRNA-based diagnostic tools for advanced fibrosis and cirrhosis in patients with chronic hepatitis B and C. Sci. Rep..

[40] Roderburg C., Urban G.W., Bettermann K., Vucur M., Zimmermann H., Schmidt S., Janssen J., Koppe C., Knolle P., Castoldi M. (2011). Micro-RNA profiling reveals a role for miR-29 in human and murine liver fibrosis. Hepatology.

[41] El-Ahwany E., Nagy F., Zoheiry M., Shemis M., Nosseir M., Taleb H.A., El Ghannam M., Atta R., Zada S. (2016). Circulating miRNAs as predictor markers for activation of hepatic stellate cells and progression of HCV-induced liver fibrosis. Electron. Physician.

[42] Cermelli S., Ruggieri A., Marrero J.A., Ioannou G.N., Beretta L. (2011). Circulating microRNAs in patients with chronic hepatitis C and non-alcoholic fatty liver disease. PLoS ONE.

[43] Markovic J., Sharma A.D., Balakrishnan A. (2020). MicroRNA-221: A fine tuner and potential biomarker of chronic liver injury. Cells.

[44] Motawi T.K., Shaker O.G., El-Maraghy S.A., Senousy M.A. (2015). Serum microRNAs as potential biomarkers for early diagnosis of hepatitis C virus-related hepatocellular carcinoma in Egyptian patients. PLoS ONE.

[45] Yoon J.S., Kim G., Lee Y.R., Park S.Y., Tak W.Y., Kweon Y.O., Park J.G., Lee H.W., Han Y.S., Ha H.T. (2018). Clinical significance of microRNA-21 expression in disease progression of patients with hepatocellular carcinoma. Biomark. Med..

[46] Waidmann O., Koberle V., Brunner F., Zeuzem S., Piiper A., Kronenberger B. (2012). Serum microRNA-122 predicts survival in patients with liver cirrhosis. PLoS ONE.

[47] Nabiel Y., Barakat G., Abed S. (2019). Serum CD64 and ascitic fluid calprotectin and microRNA-155 as potential biomarkers of spontaneous bacterial peritonitis. Eur. J. Gastroenterol. Hepatol..

[48] Lutz P., M’haimid M., Pohlmann A., Lehmann J., Jansen C., Schierwagen R., Klein S., Strassburg C.P., Spengler U., Trebicka J. (2017). MicroRNA-155 is upregulated in ascites in patients with spontaneous bacterial peritonitis. Sci. Rep..

[49] Schindler P., Kupcinskas J., Juzenas S., Skieceviciene J., Salteniene V., Schulz C., Weigt J., Malfertheiner P., Link A. (2018). Expression of microRNAs in the ascites of patients with peritoneal carcinomatosis and peritonitis. Cancer Cytopathol..

[50] Wang W., Liu R., Su Y., Li H., Xie W., Ning B. (2018). MicroRNA-21-5p mediates TGF-beta-regulated fibrogenic activation of spinal fibroblasts and the formation of fibrotic scars after spinal cord injury. Int. J. Biol. Sci..

[51] Zheng J., Zhou Z., Xu Z., Li G., Dong P., Chen Z., Lin D., Chen B., Yu F. (2015). Serum microRNA-125a-5p, a useful biomarker in liver diseases, correlates with disease progression. Mol. Med. Rep..

[52] Zhang H., Yan X.L., Guo X.X., Shi M.J., Lu Y.Y., Zhou Q.M., Chen Q.L., Hu Y.Y., Xu L.M., Huang S. (2018). MiR-27a as a predictor for the activation of hepatic stellate cells and hepatitis B virus-induced liver cirrhosis. Oncotarget.

[53] Yu F., Zhou G., Li G., Chen B., Dong P., Zheng J. (2015). Serum miR-181b is correlated with hepatitis B virus replication and disease progression in chronic hepatitis B patients. Dig. Dis. Sci..

[54] Abdel-Al A., El-Ahwany E., Zoheiry M., Hassan M., Ouf A., Abu-Taleb H., Abdel Rahim A., El-Talkawy M.D., Zada S. (2018). MiRNA-221 and miRNA-222 are promising biomarkers for progression of liver fibrosis in HCV Egyptian patients. Virus Res..

[55] Mourad L., El-Ahwany E., Zoheiry M., Abu-Taleb H., Hassan M., Ouf A., Rahim A.A., Hassanien M., Zada S. (2018). Expression analysis of liver-specific circulating microRNAs in HCV-induced hepatocellular carcinoma in Egyptian patients. Cancer Biol. Ther..

[56] Marquez R.T., Bandyopadhyay S., Wendlandt E.B., Keck K., Hoffer B.A., Icardi M.S., Christensen R.N., Schmidt W.N., McCaffrey A.P. (2010). Correlation between microRNA expression levels and clinical parameters associated with chronic hepatitis C viral infection in humans. Lab. Investig..

[57] Lee E.B., Sung P.S., Kim J.H., Park D.J., Hur W., Yoon S.K. (2020). MicroRNA-99a restricts replication of hepatitis C virus by targeting mTOR and de novo Lipogenesis. Viruses.

[58] Cabral B.C.A., Hoffmann L., Bottaro T., Costa P.F., Ramos A.L.A., Coelho H.S.M., Villela-Nogueira C.A., Urmenyi T.P., Faffe D.S., Silva R. (2020). Circulating microRNAs associated with liver fibrosis in chronic hepatitis C patients. Biochem. Biophys. Rep..

[59] Halasz T., Horvath G., Par G., Werling K., Kiss A., Schaff Z., Lendvai G. (2015). MiR-122 negatively correlates with liver fibrosis as detected by histology and FibroScan. World J. Gastroenterol..

[60] Ramachandran S., Ilias Basha H., Sarma N.J., Lin Y., Crippin J.S., Chapman W.C., Mohanakumar T. (2013). Hepatitis C virus induced miR200c down modulates FAP-1, a negative regulator of Src signaling and promotes hepatic fibrosis. PLoS ONE.

[61] Shrivastava S., Petrone J., Steele R., Lauer G.M., Di Bisceglie A.M., Ray R.B. (2013). Up-regulation of circulating miR-20a is correlated with hepatitis C virus-mediated liver disease progression. Hepatology.

[62] Ding J., Li M., Wan X., Jin X., Chen S., Yu C., Li Y. (2015). Effect of miR-34a in regulating steatosis by targeting PPARalpha expression in nonalcoholic fatty liver disease. Sci. Rep..

[63] Xin S., Zhan Q., Chen X., Xu J., Yu Y. (2020). Efficacy of serum miRNA test as a non-invasive method to diagnose nonalcoholic steatohepatitis: A systematic review and meta-analysis. BMC Gastroenterol..

[64] Gerhard G.S., Hanson A., Wilhelmsen D., Piras I.S., Still C.D., Chu X., Petrick A.T., DiStefano J.K. (2019). AEBP1 expression increases with severity of fibrosis in NASH and is regulated by glucose, palmitate, and miR-372-3p. PLoS ONE.

[65] Jia H.Y., Chen F., Chen J.Z., Wu S.S., Wang J., Cao Q.Y., Chen Z., Zhu H.H. (2014). MicroRNA expression profiles related to early stage murine concanavalin A-induced hepatitis. Cell Physiol. Biochem..

[66] Tadokoro T., Morishita A., Sakamoto T., Fujihara S., Fujita K., Mimura S., Oura K., Nomura T., Tani J., Yoneyama H. (2017). Galectin9 ameliorates fulminant liver injury. Mol. Med. Rep..

[67] Huang C., Xing X., Xiang X., Fan X., Men R., Ye T., Yang L. (2020). MicroRNAs in autoimmune liver diseases: From diagnosis to potential therapeutic targets. Biomed. Pharmacother..

[68] Afonso M.B., Rodrigues P.M., Simao A.L., Gaspar M.M., Carvalho T., Borralho P., Banales J.M., Castro R.E., Rodrigues C.M.P. (2018). MiRNA-21 ablation protects against liver injury and necroptosis in cholestasis. Cell Death Differ..

[69] Kim Y.C., Jung H., Seok S., Zhang Y., Ma J., Li T., Kemper B., Kemper J.K. (2020). MicroRNA-210 promotes bile acid-induced cholestatic liver injury by targeting mixed-lineage leukemia-4 methyltransferase in mice. Hepatology.

[70] Sakamoto T., Morishita A., Nomura T., Tani J., Miyoshi H., Yoneyama H., Iwama H., Himoto T., Masaki T. (2016). Identification of microRNA profiles associated with refractory primary biliary cirrhosis. Mol. Med. Rep..

[71] Liang D.Y., Hou Y.Q., Luo L.J., Ao L. (2016). Altered expression of miR-92a correlates with Th17 cell frequency in patients with primary biliary cirrhosis. Int. J. Mol. Med..

[72] Wang X., Wen X., Zhou J., Qi Y., Wu R., Wang Y., Kui Y., Hua R., Jin Q. (2017). MicroRNA-223 and microRNA-21 in peripheral blood B cells associated with progression of primary biliary cholangitis patients. PLoS ONE.

[73] Katsumi T., Ninomiya M., Nishina T., Mizuno K., Tomita K., Haga H., Okumoto K., Saito T., Shimosegawa T., Ueno Y. (2016). MiR-139-5p is associated with inflammatory regulation through c-FOS suppression, and contributes to the progression of primary biliary cholangitis. Lab. Investig..

[74] Wu X., Xia M., Chen D., Wu F., Lv Z., Zhan Q., Jiao Y., Wang W., Chen G., An F. (2016). Profiling of downregulated blood-circulating miR-150-5p as a novel tumor marker for cholangiocarcinoma. Tumor Biol..

[75] Friedrich K., Baumann C., Wannhoff A., Rupp C., Mehrabi A., Weiss K.H., Gotthardt D.N. (2018). Serum miRNA-122 is an independent biomarker of survival in patients with primary sclerosing cholangitis. J. Gastrointest. Liver Dis..

[76] Bernuzzi F., Marabita F., Lleo A., Carbone M., Mirolo M., Marzioni M., Alpini G., Alvaro D., Boberg K.M., Locati M. (2016). Serum microRNAs as novel biomarkers for primary sclerosing cholangitis and cholangiocarcinoma. Clin. Exp. Immunol..

[77] Hayes C.N., Chayama K. (2016). MicroRNAs as biomarkers for liver disease and hepatocellular carcinoma. Int. J. Mol. Sci..

[78] Ma L., Yang X., Wei R., Ye T., Zhou J.K., Wen M., Men R., Li P., Dong B., Liu L. (2018). MicroRNA-214 promotes hepatic stellate cell activation and liver fibrosis by suppressing Sufu expression. Cell Death Dis..

[79] Rogler C.E., Matarlo J.S., Kosmyna B., Fulop D., Rogler L.E. (2017). Knockdown of miR-23, miR-27, and miR-24 alters fetal liver development and blocks fibrosis in mice. Gene Expr..

[80] Chen J., Yu Y., Li S., Liu Y., Zhou S., Cao S., Yin J., Li G. (2017). MicroRNA-30a ameliorates hepatic fibrosis by inhibiting Beclin1-mediated autophagy. J. Cell. Mol. Med..

[81] Matsumoto Y., Itami S., Kuroda M., Yoshizato K., Kawada N., Murakami Y. (2016). MiR-29a assists in preventing the activation of human stellate cells and promotes recovery from liver fibrosis in mice. Mol. Ther..

[82] Lin H.Y., Wang F.S., Yang Y.L., Huang Y.H. (2019). MicroRNA-29a suppresses CD36 to ameliorate high fat diet-induced steatohepatitis and liver fibrosis in mice. Cells.

[83] Huang Y.H., Kuo H.C., Yang Y.L., Wang F.S. (2019). MicroRNA-29a is a key regulon that regulates BRD4 and mitigates liver fibrosis in mice by inhibiting hepatic stellate cell activation. Int. J. Med. Sci..

[84] Feili X., Wu S., Ye W., Tu J., Lou L. (2018). MicroRNA-34a-5p inhibits liver fibrosis by regulating TGF-beta1/Smad3 pathway in hepatic stellate cells. Cell Biol. Int..

[85] Calvente C.J., Tameda M., Johnson C.D., Del Pilar H., Lin Y.C., Adronikou N., De Mollerat Du Jeu X., Llorente C., Boyer J., Feldstein A.E. (2019). Neutrophils contribute to spontaneous resolution of liver inflammation and fibrosis via microRNA-223. J. Clin. Investig..

[86] Wei S., Wang Q., Zhou H., Qiu J., Li C., Shi C., Zhou S., Liu R., Lu L. (2019). MiR-455-3p alleviates hepatic stellate cell activation and liver fibrosis by suppressing HSF1 expression. Mol. Ther. Nucleic Acids.

[87] Hu Z., Li L., Ran J., Chu G., Gao H., Guo L., Chen J. (2019). MiR-125b acts as anti-fibrotic therapeutic target through regulating Gli3 In Vivo and In Vitro. Ann. Hepatol..

[88] Hyun J., Wang S., Kim J., Rao K.M., Park S.Y., Chung I., Ha C.S., Kim S.W., Yun Y.H., Jung Y. (2016). MicroRNA-378 limits activation of hepatic stellate cells and liver fibrosis by suppressing Gli3 expression. Nat. Commun..

[89] Li L., Zhang L., Zhao X., Cao J., Li J., Chu G. (2019). Downregulation of miR-152 contributes to the progression of liver fibrosis via targeting Gli3 In Vivo and In Vitro. Exp. Ther. Med..

[90] Zhu H., Fan G.C. (2012). Role of microRNAs in the reperfused myocardium towards post-infarct remodelling. Cardiovasc. Res..

[91] Fish J.E., Santoro M.M., Morton S.U., Yu S., Yeh R.F., Wythe J.D., Ivey K.N., Bruneau B.G., Stainier D.Y., Srivastava D. (2008). MiR-126 regulates angiogenic signaling and vascular integrity. Dev. Cell.

[92] Satishchandran A., Ambade A., Rao S., Hsueh Y.C., Iracheta-Vellve A., Tornai D., Lowe P., Gyongyosi B., Li J., Catalano D. (2018). MicroRNA 122, regulated by GRLH2, protects livers of mice and patients from ethanol-induced liver disease. Gastroenterology.

[93] Li M., He Y., Zhou Z., Ramirez T., Gao Y., Gao Y., Ross R.A., Cao H., Cai Y., Xu M. (2017). MicroRNA-223 ameliorates alcoholic liver injury by inhibiting the IL-6-p47(phox)-oxidative stress pathway in neutrophils. Gut.

[94] Bala S., Csak T., Saha B., Zatsiorsky J., Kodys K., Catalano D., Satishchandran A., Szabo G. (2016). The pro-inflammatory effects of miR-155 promote liver fibrosis and alcohol-induced steatohepatitis. J. Hepatol..

[95] Saikia P., Bellos D., McMullen M.R., Pollard K.A., de la Motte C., Nagy L.E. (2017). MicroRNA 181b-3p and its target importin alpha5 regulate toll-like receptor 4 signaling in Kupffer cells and liver injury in mice in response to ethanol. Hepatology.

[96] Yin H., Hu M., Zhang R., Shen Z., Flatow L., You M. (2012). MicroRNA-217 promotes ethanol-induced fat accumulation in hepatocytes by down-regulating SIRT1. J. Biol. Chem..

[97] Lai C.Y., Yeh K.Y., Lin C.Y., Hsieh Y.W., Lai H.H., Chen J.R., Hsu C.C., Her G.M. (2021). MicroRNA-21 plays multiple oncometabolic roles in the process of NAFLD-related hepatocellular carcinoma via PI3K/AKT, TGF-beta, and STAT3 signaling. Cancers.

[98] Yang Y.L., Kuo H.C., Wang F.S., Huang Y.H. (2019). MicroRNA-29a disrupts DNMT3b to ameliorate diet-induced non-alcoholic steatohepatitis in mice. Int. J. Mol. Sci..

[99] Chai C., Cox B., Yaish D., Gross D., Rosenberg N., Amblard F., Shemuelian Z., Gefen M., Korach A., Tirosh O. (2020). Agonist of RORA attenuates nonalcoholic fatty liver progression in mice via up-regulation of MicroRNA 122. Gastroenterology.

[100] Zhang Z., Wen H., Peng B., Weng J., Zeng F. (2020). Downregulated microRNA-129-5p by long non-coding RNA NEAT1 upregulates PEG3 expression to aggravate non-alcoholic steatohepatitis. Front. Genet..

[101] Riaz F., Chen Q., Lu K., Osoro E.K., Wu L., Feng L., Zhao R., Yang L., Zhou Y., He Y. (2021). Inhibition of miR-188-5p alleviates hepatic fibrosis by significantly reducing the activation and proliferation of HSCs through PTEN/PI3K/AKT pathway. J. Cell. Mol. Med..

[102] He Y., Rodrigues R.M., Wang X., Seo W., Ma J., Hwang S., Fu Y., Trojnar E., Matyas C., Zhao S. (2021). Neutrophil-to-hepatocyte communication via LDLR-dependent miR-223-enriched extracellular vesicle transfer ameliorates nonalcoholic steatohepatitis. J. Clin. Investig..

[103] Benito-Vicente A., Uribe K.B., Rotllan N., Ramirez C.M., Jebari-Benslaiman S., Goedeke L., Canfran-Duque A., Galicia-Garcia U., Saenz De Urturi D., Aspichueta P. (2020). MiR-27b modulates insulin signaling in hepatocytes by regulating insulin receptor expression. Int. J. Mol. Sci..

[104] Ke Q.H., Chen H.Y., He Z.L., Lv Z., Xu X.F., Qian Y.G., Zheng S.S. (2019). Silencing of microRNA-375 affects immune function in mice with liver failure by upregulating astrocyte elevated gene-1 through reducing apoptosis of Kupffer cells. J. Cell Biochem..

[105] Wang Y.Q., Lan Y.Y., Guo Y.C., Yuan Q.W., Liu P. (2019). Down-regulation of microRNA-138 improves immunologic function via negatively targeting p53 by regulating liver macrophage in mice with acute liver failure. Biosci. Rep..

[106] Lu Z., Liu J., Liu X., Huang E., Yang J., Qian J., Zhang D., Liu R., Chu Y. (2018). MicroRNA 15a/16-1 suppresses aryl hydrocarbon receptor-dependent interleukin-22 secretion in CD4(+) T cells and contributes to immune-mediated organ injury. Hepatology.

[107] Su K., Wang Q., Qi L., Hua D., Tao J., Mangan C.J., Lou Y., Li L. (2016). MicroRNA-674-5p/5-LO axis involved in autoimmune reaction of Concanavalin A-induced acute mouse liver injury. Toxicol. Lett..

[108] Tu H., Chen D., Cai C., Du Q., Lin H., Pan T., Sheng L., Xu Y., Teng T., Tu J. (2020). MicroRNA-143-3p attenuated development of hepatic fibrosis in autoimmune hepatitis through regulation of TAK1 phosphorylation. J. Cell. Mol. Med..

[109] Yang F., Lou G., Zhou X., Zheng M., He J., Chen Z. (2014). MicroRNA-223 acts as an important regulator to Kupffer cells activation at the early stage of Con A-induced acute liver failure via AIM2 signaling pathway. Cell. Physiol. Biochem..

[110] Marzioni M., Agostinelli L., Candelaresi C., Saccomanno S., De Minicis S., Maroni L., Mingarelli E., Rychlicki C., Trozzi L., Banales J.M. (2014). Activation of the developmental pathway neurogenin-3/microRNA-7a regulates cholangiocyte proliferation in response to injury. Hepatology.

[111] Fernandez-Ramos D., Fernandez-Tussy P., Lopitz-Otsoa F., Gutierrez-de-Juan V., Navasa N., Barbier-Torres L., Zubiete-Franco I., Simon J., Fernandez A.F., Arbelaiz A. (2018). MiR-873-5p acts as an epigenetic regulator in early stages of liver fibrosis and cirrhosis. Cell Death Dis..

[112] Hall C., Ehrlich L., Meng F., Invernizzi P., Bernuzzi F., Lairmore T.C., Alpini G., Glaser S. (2017). Inhibition of microRNA-24 increases liver fibrosis by enhanced menin expression in Mdr2(-/-) mice. J. Surg. Res..

[113] Chen L., Chen R., Kemper S., Charrier A., Brigstock D.R. (2015). Suppression of fibrogenic signaling in hepatic stellate cells by Twist1-dependent microRNA-214 expression: Role of exosomes in horizontal transfer of Twist1. Am. J. Physiol. Gastrointest. Liver Physiol..

[114] Chen L., Chen R., Velazquez V.M., Brigstock D.R. (2016). Fibrogenic signaling is suppressed in hepatic stellate cells through targeting of connective tissue growth factor (CCN2) by cellular or exosomal microRNA-199a-5p. Am. J. Pathol..

[115] Povero D., Panera N., Eguchi A., Johnson C.D., Papouchado B.G., de Araujo Horcel L., Pinatel E.M., Alisi A., Nobili V., Feldstein A.E. (2015). Lipid-induced hepatocyte-derived extracellular vesicles regulate hepatic stellate cell via microRNAs targeting PPAR-gamma. Cell. Mol. Gastroenterol. Hepatol..

[116] Brandon-Warner E., Feilen N.A., Culberson C.R., Field C.O., deLemos A.S., Russo M.W., Schrum L.W. (2016). Processing of miR17-92 cluster in hepatic stellate cells promotes hepatic fibrogenesis during alcohol-induced injury. Alcohol Clin. Exp. Res..

[117] Geiger A., Walker A., Nissen E. (2015). Human fibrocyte-derived exosomes accelerate wound healing in genetically diabetic mice. Biochem. Biophys. Res. Commun..

[118] Lou G., Yang Y., Liu F., Ye B., Chen Z., Zheng M., Liu Y. (2017). MiR-122 modification enhances the therapeutic efficacy of adipose tissue-derived mesenchymal stem cells against liver fibrosis. J. Cell. Mol. Med..

[119] Chen L., Chen R., Kemper S., Cong M., You H., Brigstock D.R. (2018). Therapeutic effects of serum extracellular vesicles in liver fibrosis. J. Extracell. Vesicles.

[120] Guo C.J., Pan Q., Xiong H., Qiao Y.Q., Bian Z.L., Zhong W., Sheng L., Li H., Shen L., Hua J. (2014). Therapeutic potential of microRNA: A new target to treat intrahepatic portal hypertension?. Biomed. Res. Int..

[121] Breitkopf K., Godoy P., Ciuclan L., Singer M.V., Dooley S. (2006). TGF-beta/Smad signaling in the injured liver. Z. Gastroenterol..

[122] Liang J., Deng X., Lin Z.X., Zhao L.C., Zhang X.L. (2009). Attenuation of portal hypertension by natural taurine in rats with liver cirrhosis. World J. Gastroenterol..

[123] Thabut D., Shah V. (2010). Intrahepatic angiogenesis and sinusoidal remodeling in chronic liver disease: New targets for the treatment of portal hypertension?. J. Hepatol..

[124] Chawla Y.K., Bodh V. (2015). Portal vein thrombosis. J. Clin. Exp. Hepatol..

[125] Vemuganti R., Silva V.R., Mehta S.L., Hazell A.S. (2014). Acute liver failure-induced hepatic encephalopathy s associated with changes in microRNA expression rofiles in cerebral cortex of the mouse [corrected]. Metab. Brain Dis..

[126] Baker L., Lanz B., Andreola F., Ampuero J., Wijeyesekera A., Holmes E., Deutz N. (2016). New technologies—New insights into the pathogenesis of hepatic encephalopathy. Metab. Brain Dis..

[127] Oenarto J., Karababa A., Castoldi M., Bidmon H.J., Gorg B., Haussinger D. (2016). Ammonia-induced miRNA expression changes in cultured rat astrocytes. Sci. Rep..

[128] Fattovich G., Brollo L., Giustina G., Noventa F., Pontisso P., Alberti A., Realdi G., Ruol A. (1991). Natural history and prognostic factors for chronic hepatitis type B. Gut.

[129] Kanda T., Goto T., Hirotsu Y., Moriyama M., Omata M. (2019). Molecular mechanisms driving progression of liver cirrhosis towards hepatocellular carcinoma in chronic hepatitis B and C infections: A review. Int. J. Mol. Sci..

[130] Dienstag J.L., Goldin R.D., Heathcote E.J., Hann H.W., Woessner M., Stephenson S.L., Gardner S., Gray D.F., Schiff E.R. (2003). Histological outcome during long-term lamivudine therapy. Gastroenterology.

[131] Hadziyannis S.J., Tassopoulos N.C., Heathcote E.J., Chang T.T., Kitis G., Rizzetto M., Marcellin P., Lim S.G., Goodman Z., Ma J. (2006). Long-term therapy with adefovir dipivoxil for HBeAg-negative chronic hepatitis B for up to 5 years. Gastroenterology.

[132] Jiang X., Kanda T., Wu S., Nakamura M., Miyamura T., Nakamoto S., Banerjee A., Yokosuka O. (2014). Regulation of microRNA by hepatitis B virus infection and their possible association with control of innate immunity. World J. Gastroenterol..

[133] Hayes C.N., Akamatsu S., Tsuge M., Miki D., Akiyama R., Abe H., Ochi H., Hiraga N., Imamura M., Takahashi S. (2012). Hepatitis B virus-specific miRNAs and Argonaute2 play a role in the viral life cycle. PLoS ONE.

[134] Arataki K., Hayes C.N., Akamatsu S., Akiyama R., Abe H., Tsuge M., Miki D., Ochi H., Hiraga N., Imamura M. (2013). Circulating microRNA-22 correlates with microRNA-122 and represents viral replication and liver injury in patients with chronic hepatitis B. J. Med. Virol..

[135] Liu W.H., Yeh S.H., Chen P.J. (2011). Role of microRNAs in hepatitis B virus replication and pathogenesis. Biochim. Biophys. Acta.

[136] Thakral S., Ghoshal K. (2015). MiR-122 is a unique molecule with great potential in diagnosis, prognosis of liver disease, and therapy both as miRNA mimic and antimir. Curr. Gene Ther..

[137] Ji F., Yang B., Peng X., Ding H., You H., Tien P. (2011). Circulating microRNAs in hepatitis B virus-infected patients. J. Viral Hepat..

[138] Cheong J.Y., Shin H.D., Kim Y.J., Cho S.W. (2013). Association of polymorphism in MicroRNA 219-1 with clearance of hepatitis B virus infection. J. Med. Virol..

[139] Qiu L., Fan H., Jin W., Zhao B., Wang Y., Ju Y., Chen L., Chen Y., Duan Z., Meng S. (2010). MiR-122-induced down-regulation of HO-1 negatively affects miR-122-mediated suppression of HBV. Biochem. Biophys. Res. Commun..

[140] Qiao D.D., Yang J., Lei X.F., Mi G.L., Li S.L., Li K., Xu C.Q., Yang H.L. (2017). Expression of microRNA-122 and microRNA-22 in HBV-related liver cancer and the correlation with clinical features. Eur. Rev. Med. Pharmacol. Sci..

[141] Coppola N., Onorato L., Panella M., de Stefano G., Mosca N., Minichini C., Messina V., Potenza N., Starace M., Alessio L. (2018). Correlation between the hepatic expression of human microRNA hsa-miR-125a-5p and the progression of fibrosis in patients with overt and occult HBV infection. Front. Immunol..

[142] Singh A.K., Rooge S.B., Varshney A., Vasudevan M., Bhardwaj A., Venugopal S.K., Trehanpati N., Kumar M., Geffers R., Kumar V. (2018). Global microRNA expression profiling in the liver biopsies of hepatitis B virus-infected patients suggests specific microRNA signatures for viral persistence and hepatocellular injury. Hepatology.

[143] Shaker O.G., Senousy M.A. (2017). Serum microRNAs as predictors for liver fibrosis staging in hepatitis C virus-associated chronic liver disease patients. J. Viral Hepat..

[144] Kunden R.D., Khan J.Q., Ghezelbash S., Wilson J.A. (2020). The role of the liver-specific microRNA, miRNA-122 in the HCV replication cycle. Int. J. Mol. Sci..

[145] Gao B., Bataller R. (2011). Alcoholic liver disease: Pathogenesis and new therapeutic targets. Gastroenterology.

[146] Bala S., Petrasek J., Mundkur S., Catalano D., Levin I., Ward J., Alao H., Kodys K., Szabo G. (2012). Circulating microRNAs in exosomes indicate hepatocyte injury and inflammation in alcoholic, drug-induced, and inflammatory liver diseases. Hepatology.

[147] Bala S., Marcos M., Kodys K., Csak T., Catalano D., Mandrekar P., Szabo G. (2011). Up-regulation of microRNA-155 in macrophages contributes to increased tumor necrosis factor α (TNFα) production via increased mRNA half-life in alcoholic liver disease. J. Biol. Chem..

[148] Ford E.S., Giles W.H., Dietz W.H. (2002). Prevalence of the metabolic syndrome among US adults: Findings from the third National Health and Nutrition Examination Survey. JAMA.

[149] Kamada Y., Ono M., Hyogo H., Fujii H., Sumida Y., Yamada M., Mori K., Tanaka S., Maekawa T., Ebisutani Y. (2017). Use of Mac-2 binding protein as a biomarker for nonalcoholic fatty liver disease diagnosis. Hepatol. Commun..

[150] Dowman J.K., Tomlinson J.W., Newsome P.N. (2010). Pathogenesis of non-alcoholic fatty liver disease. QJM.

[151] Chen W., Zhang J., Fan H.N., Zhu J.S. (2018). Function and therapeutic advances of chemokine and its receptor in nonalcoholic fatty liver disease. Ther. Adv. Gastroenterol..

[152] Peverill W., Powell L.W., Skoien R. (2014). Evolving concepts in the pathogenesis of NASH: Beyond steatosis and inflammation. Int. J. Mol. Sci..

[153] Torres J.L., Novo-Veleiro I., Manzanedo L., Alvela-Suarez L., Macias R., Laso F.J., Marcos M. (2018). Role of microRNAs in alcohol-induced liver disorders and non-alcoholic fatty liver disease. World J. Gastroenterol..

[154] Katsura A., Morishita A., Iwama H., Tani J., Sakamoto T., Tatsuta M., Toyota Y., Fujita K., Kato K., Maeda E. (2015). MicroRNA profiles following metformin treatment in a mouse model of non-alcoholic steatohepatitis. Int. J. Mol. Med..

[155] de Boer Y.S., van Gerven N.M., Zwiers A., Verwer B.J., van Hoek B., van Erpecum K.J., Beuers U., van Buuren H.R., Drenth J.P., den Ouden J.W. (2014). Genome-wide association study identifies variants associated with autoimmune hepatitis type 1. Gastroenterology.

[156] Ueno K., Aiba Y., Hitomi Y., Shimoda S., Nakamura H., Gervais O., Kawai Y., Kawashima M., Nishida N., Kohn S.S. (2020). Integrated GWAS and mRNA microarray analysis identified IFNG and CD40L as the central upstream regulators in primary biliary cholangitis. Hepatol. Commun..

[157] Krawitt E.L. (2006). Autoimmune hepatitis. N. Engl. J. Med..

[158] European Association for the Study of the Liver (2015). EASL Clinical Practice Guidelines: Autoimmune hepatitis. J. Hepatol..

[159] Tomiyama T., Yang G.X., Zhao M., Zhang W., Tanaka H., Wang J., Leung P.S., Okazaki K., He X.S., Lu Q. (2017). The modulation of co-stimulatory molecules by circulating exosomes in primary biliary cirrhosis. Cell. Mol. Immunol..

